# The Role of Kinin Receptors in Preventing Neuroinflammation and Its Clinical Severity during Experimental Autoimmune Encephalomyelitis in Mice

**DOI:** 10.1371/journal.pone.0027875

**Published:** 2011-11-22

**Authors:** Rafael C. Dutra, Daniela F. P. Leite, Allisson F. Bento, Marianne N. Manjavachi, Eliziane S. Patrício, Cláudia P. Figueiredo, João B. Pesquero, João B. Calixto

**Affiliations:** 1 Department of Pharmacology, Centre of Biological Sciences, Universidade Federal de Santa Catarina, Florianópolis, Santa Catarina, Brazil; 2 Department of Biophysics, Universidade Federal de São Paulo, São Paulo, Brazil; Washington University, United States of America

## Abstract

**Background:**

Multiple sclerosis (MS) is a demyelinating and neuroinflammatory disease of the human central nervous system (CNS). The expression of kinins is increased in MS patients, but the underlying mechanisms by which the kinin receptor regulates MS development have not been elucidated.

**Methodology/Principal Findings:**

Experimental autoimmune encephalomyelitis (EAE) was induced in female C57BL/6 mice by immunization with MOG_35–55_ peptide emulsified in complete Freund's adjuvant and injected with pertussis toxin on day 0 and day 2. Here, we report that blockade of the B_1_R in the induction phase of EAE markedly suppressed its progression by interfering with the onset of the immune response. Furthermore, B_1_R antagonist suppressed the production/expression of antigen-specific T_H_1 and T_H_17 cytokines and transcription factors, both in the periphery and in the CNS. In the chronic phase of EAE, the blockade of B_1_R consistently impaired the clinical progression of EAE. Conversely, administration of the B_1_R agonist in the acute phase of EAE suppressed disease progression and inhibited the increase in permeability of the blood-brain barrier (BBB) and any further CNS inflammation. Of note, blockade of the B_2_R only showed a moderate impact on all of the studied parameters of EAE progression.

**Conclusions/Significance:**

Our results strongly suggest that kinin receptors, mainly the B_1_R subtype, play a dual role in EAE progression depending on the phase of treatment through the lymphocytes and glial cell-dependent pathways.

## Introduction

Multiple sclerosis (MS) is the most common inflammatory demyelinating disease of the central nervous system (CNS) that cause neurological disability in young adults, affecting about two million people worldwide [1], [2]. The hallmarks of MS include neuronal loss, axonal injury and atrophy of the CNS, due to a progressive inflammatory reaction involving both the adaptive and the innate immune system [3], [4], [5]. During the course of MS, autoreactive T cells activated in the periphery by viral or infectious antigens, which show molecular similarity to the CNS antigen [6], differentiate into T_H_1 or T_H_17 cells, migrate across the blood-brain barrier (BBB) and successively induce inflammatory lesions distributed throughout the CNS [2].

The CNS of mammals contains all of the components of the kallikrein-kinin system [7] and accumulating evidence suggests that these components are altered in neurodegenerative processes [8], [9], [10]. The biological activities of kinin are mediated via two G-protein-coupled receptors, named the B_1_ (B_1_R) and B_2_ (B_2_R) receptors. The B_2_R is constitutively expressed throughout central and peripheral tissues, while the B_1_R is normally up-regulated following inflammatory, infectious or traumatic stimuli, exerting a critical role in several chronic diseases [11], [12].

Recent reports demonstrated the involvement of the kinins and their receptors in MS and the experimental autoimmune encephalomyelitis (EAE) model [13], [14], [15]. For instance, high levels of the kallikrein-kinin components, namely des-Arg^9^-bradykinin (DABK), bradykinin, kallikrein-1 and kallikrein-6, as well as low-molecular-weight kininogens (KNGL), have been found in the CNS tissue and cerebrospinal fluid from both animals with EAE and MS patients [16], [17].

Experiments carried out with B_2_R-knockout mice showed that the clinical parameters of MOG_35–55_-induced EAE are reduced via the modulation of leukocyte recruitment into the CNS [14]; however, the participation of B_2_R seems to be less important than B_1_R in the development of EAE [15], [17]. It was recently shown that B_1_ mRNA expression positively correlated with the expanded disability status scale (EDSS) index and the occurrence of clinical relapse in patients with MS [13]. In addition, B_1_R was found to be up-regulated in both the brain endothelial cells [18] and peripheral T lymphocyte cells in these patients [13].

It is widely accepted that, following their activation, both B_1_R and B_2_R induce inflammation via the release of pro-inflammatory cytokines and increased vascular permeability [11], [19]. In marked contrast to this, a recent paper suggested exactly the opposite, i.e. that the activation of B_1_R protects against encephalitogenic T lymphocyte recruitment to the CNS [17]. For this reason, we hypothesized that kinin receptor, mainly B_1_R subtypes, could display a dual role in EAE by acting at different phases of disease progression. We further examined this hypothesis by using B_1_ and B_2_-knockout mice in conjugation with a kinin selective agonist or antagonist at different time points after the induction of EAE.

## Results

### Dominant role of kinin B_1_R in the induction phase of EAE

**Figure 1:**
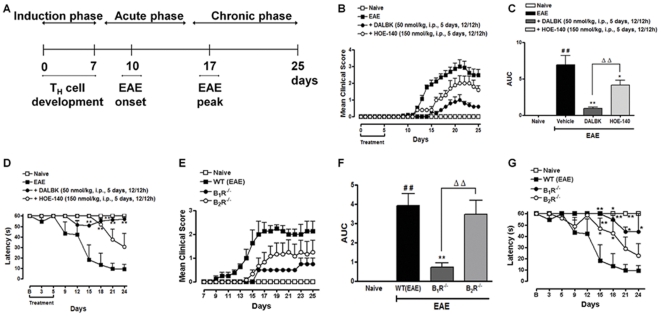
Kinin B_1_R inhibition or deletion in the disease induction phase attenuated the development of EAE and reduced the inflammatory response in the CNS 25 days post-immunization (p.i.). Animals were immunized with MOG_35–55_ peptide/CFA and pertussis toxin. (A) Schematic representation of EAE progression. Day 0 to 7: induction phase; day 7 to 15: acute phase and day 15 to 25: chronic phase of the disease. EAE: experimental autoimmune encephalomyelitis; T_H_: CD4^+^ T helper lymphocytes. The clinical score (B, E), area under the curve (AUC) (C, F) and locomotor activity (D, G) were analyzed in the naive group, the control group (EAE), in mice pre-treated with the selective kinin B_1_R antagonist DALBK (50 nmol/kg), in mice pre-treated with the selective kinin B_2_R antagonist HOE-140 (50 nmol/kg), in B_1_R^−/−^ knockout mice and in B_2_R^−/−^ knockout mice 25 days p.i. The antagonists were administered intraperitoneally (i.p.), twice a day (12/12 h), for 5 days (day 0–5). The results of clinical score are expressed as mean or as the AUC. Data are presented as mean ± SEM of six to nine mice/group and are representative of three independent experiments. ^#^ *P*<0.05 and ^##^ *P*<0.001 versus the naive group, *P<0.05 and **P<0.001 versus the EAE group, ^Δ^P<0.05 and ^ΔΔ^P<0.001 versus the DALBK treatment or B_1_R^−/−^ mice (one-way ANOVA with the Newmann-Keuls post-hoc test).

Initially, in order to investigate the role of kinin receptors on the EAE induction phase, we induced EAE by subcutaneous injections of MOG_35–55_ in complete Freund's adjuvant (CFA), and pertussis toxin injections. By using this protocol, MOG-reactive T cells begin to accumulate in regional lymph nodes on day 7, and mice begin to develop clinical signs between days 10 and 12, with a peak at around day 17 [20]. Therefore, we defined days 0 to 7 as the induction phase, days 7–15 as the acute phase and days 15–25 as the chronic phase of the disease (see scheme in Fig. 1A). Our results showed that the EAE control group developed a first relapse with subsequent chronic disease phase, characterized by manifestation of an ascending paralysis starting from day 13 to day 19 following immunization (Fig. 1B–G). Interestingly, in the induction phase of EAE, the blockade of B_1_R by DALBK (50 nmol/kg, i.p., twice a day, days 0–5) significantly reduced disease severity when compared to the EAE control group (Fig. 1B–D). Importantly, the difference in the degree of disease severity persisted until the end of our study at day 25. In line with our findings for the DALBK treatment, the genetic deletion of B_1_R also drastically reduced the clinical score of EAE and delayed the disease onset (Fig. 1E–G). In contrast, preventive treatment with the B_2_R antagonist HOE-140 (150 nmol/kg, i.p., twice a day, days 0–5) (Fig. 1B–D), or the use of mice with a deletion of B_2_R, only resulted in a moderate inhibition (Fig. 1E–G). These data suggest a dominant role of kinin B_1_R in reducing EAE severity during the induction phase.

### Kinin B_1_R inhibition or its genetic deletion in the disease induction phase decrease the neuroinflammatory response and myelin loss in the spinal cord

**Figure 2:**
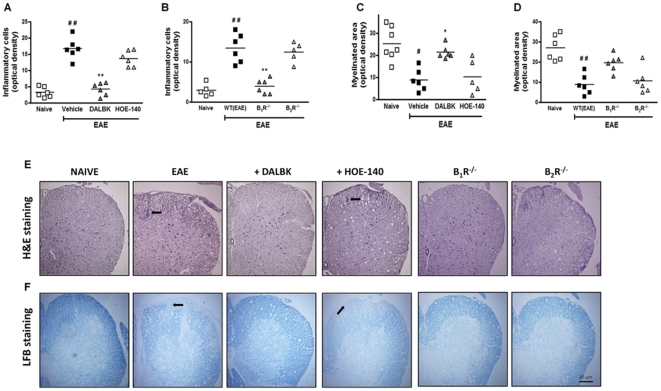
Kinin B_1_R inhibition or genetic deletion decreased the level of inflammatory cell infiltration and the demyelination area in experimental EAE. The lumbar spinal cords were histologically analyzed on day 25 p.i. in the different experimental groups for inflammation by H&E staining (A, B, E) and for demyelination by luxol fast blue staining (C, D, F). The degree of inflammatory infiltrates and demyelination was quantified from an average of four ocular field 5-µm sections of lumbar spinal cord white matter transverse sections per mouse for a total of six to nine mice/group in the naive group, the control group (EAE), in mice pre-treated with the B_1_R antagonist DALBK (50 nmol/kg), in mice pre-treated with the B_2_R antagonist HOE-140 (150 nmol/kg), in B_1_R^−/−^ knockout mice and in B_2_R^−/−^ knockout mice. Scale bar corresponds to 25 µm and applies throughout. Data are presented as mean ± SEM of six to nine mice/group and are representative of three independent experiments. ^#^P<0.05 and ^##^P<0.001 versus the naive group, *P<0.05 and **P<0.001 versus the EAE group (one-way ANOVA with the Newmann-Keuls post-hoc test).

**Figure 3:**
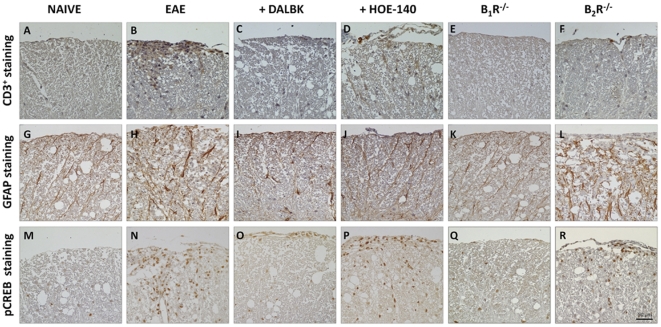
The blockade of kinin B_1_R in the disease induction phase by pharmacological treatment or genetic deletion ameliorated EAE pathology. The spinal lumbar cords obtained on the 25^th^ day after immunization from the different experimental groups were processed for immunohistochemistry assays: T cell infiltration by CD3 immunoreactivity (A–F); astrocytes activation by GFAP immunoreactivity (G–L); and CREB phosphorylation (M–R). Specifically, four 5-µm sections of lumbar spinal cord white matter (six to nine mice/group)≈150 µm apart were obtained between L4 and L6 from the naive group (A, G and M), the EAE group (B, H and N), from mice pre-treated with the B_1_R antagonist DALBK (50 nmol/kg) (C, I and O), from mice pre-treated with the B_2_R antagonist HOE-140 (150 nmol/kg) (D, J and P) and from mice deficient in B_1_R (E, K and Q) and B_2_R (F, L and R). The antagonists were administered i.p. twice a day (12/12 h), during day 0–5 p.i. Representative sections from three independent experiments are shown. Scale bar corresponds to 25 µm and applies throughout.

The hallmarks of MS include multifocal perivascular T-lymphocytes, macrophages and activated microglia infiltrates in the CNS, which induce oligodendrocyte loss and demyelination [2], [21]. In this set of experiments, we assessed the intensity of inflammatory cell infiltrates in the naive group, the control group (EAE), in mice pre-treated with the B_1_R antagonist DALBK (50 nmol/kg), in mice pre-treated with the B_2_R antagonist HOE-140 (150 nmol/kg), as well as in B_1_R^−/−^ mice and in B_2_R^−/−^ mice after 25 days post-immunization. It was found that the number of inflammatory foci was significantly decreased in B_1_R^−/−^ mice and in mice pre-treated with DALBK (*P*<0.01) (Fig. 2A, B, E). In order to further evaluate whether or not reduced inflammation in the CNS in the absence of B_1_R could preserve tissue integrity, we investigated the loss of myelin, the number of T lymphocytes and reactive astrogliosis. The demyelination index (Fig. 2C, D, F), number of CD3^+^ T cells (Fig. 3A–F) and GFAP immunoreactivity (Fig. 3G–L) were all strikingly reduced when assessed 25 days post-immunization in DALBK-treated animals during induction phase of EAE and in B_1_R^−/−^ mice. In contrast, in mice pre-treated with HOE-140 (150 nmol/kg, i.p., days 0–5) and in B_2_R^−/−^ mice, these parameters showed no significant changes (*P*>0.05) (Fig. 2A–F and Fig. 3A–L). Once again, these data indicate that kinin B_1_R shows a dominant effect on EAE neuroinflammation.

Recent studies also suggested that, at the peak of EAE, the transcription factor cyclic AMP response element-binding protein (CREB) is highly phosphorylated in the spinal cord [22], contributing to the activation of macrophages and microglia, up-regulating the MHC and co-stimulatory molecules [22], [23]. Notably, pre-treatment with the B_1_R antagonist (induction phase of EAE) or its genetic depletion resulted in a marked reduction in CREB activation at the lumbar white/grey matter of the spinal cord (Fig. 3M–R), according to the assessment 25 days post-immunization. Pre-treatment with HOE-140 or the genetic deletion of B_2_R also significantly prevented the induction of CREB phosphorylation (*P*<0.01) (Fig. 3M–R). However, the blockade of B_2_R did not significantly reduce the demyelination area nor the inflammatory cell infiltrates, suggesting that CREB phosphorylation by itself is not a limiting event in the recovery of animals with EAE.

### Blockade of kinin B_1_R in the EAE induction phase limits CD4^+^ activation/expansion and effector cytokine production in the peripheral lymphoid tissue

**Figure 4:**
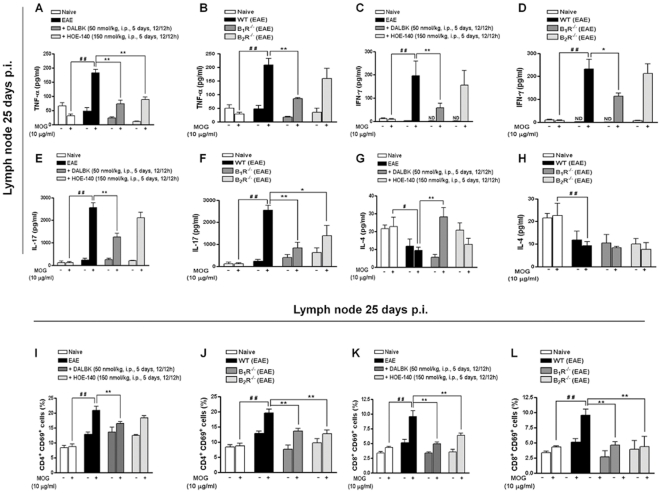
Kinin B_1_R inhibition or its deletion reduced the production of inflammatory cytokines and the activation of isolated CD4^+^ T lymphocytes. Lymphocytes (1×10^6^/well) obtained 25 days after immunization from the naive group, the control group (EAE), from mice pre-treated with the B_1_R antagonist DALBK (50 nmol/kg), from mice pre-treated with the B_2_R antagonist HOE-140 (50 nmol/kg), from B_1_R^−/−^mice and from B_2_R^−/−^ mice were cultured in the presence or absence of MOG_35–55_ (10 µg/ml) for 3 days; the supernatants were collected and measured for the concentrations of TNF-α (A, B), IFN-γ (C, D), IL-17 (E, F) and IL-4 (G, H) using ELISA assays. After culture supernatants were collected, the cells were analyzed by flow cytometry for CD4^+^CD69^+^ (I, J) and CD8^+^CD69^+^ (K, L) T cells. Each column represents the mean ± SEM of six to nine mice per group and is representative of three independent experiments. ^#^P<0.05, ^##^P<0.001 versus the naive group, *P<0.05, **P<0.001 versus the EAE group (one-way ANOVA with the Newmann-Keuls post-hoc test).

Several pieces of evidence now suggest that the extent of CNS damage in EAE is associated with peripheral T cell activation [5]. We next assessed whether B_1_R or B_2_R exert a role on peripheral lymphocyte activation. Lymph node (LN) obtained from B_1_R^−/−^ mice, B_2_R^−/−^ mice or mice previously treated with B_1_R or B_2_R antagonists were re-stimulated with MOG and analyzed for cytokine production and CD69 expression, a major T cell activation marker. We found a pronounced reduction in TNF-α (Fig. 4 A,B), IFN-γ (Fig. 4 C,D) and IL-17 (Fig. 4 E,F) levels produced by MOG-reactive LN cells isolated from mice pre-treated with DALBK and from B_1_R^−/−^ mice. In addition, pre-treatment with HOE-140 resulted in a discrete inhibition of TNF-α in LN cells (Fig. 4 A,B). Of great relevance, the blockade of B_1_R in the induction phase restored IL-4 levels in LN after *in vitro* re-stimulation with MOG (Fig. 4 G,H), suggesting that the inhibition of B_1_R may cause a shift from T_H_1/T_H_17 towards the T_H_2 phenotype. Notably, T CD4^+^ and T CD8^+^ MOG-reactive cells LN from B_1_R^−/−^, B_2_R^−/−^ mice and from mice pre-treated with DALBK showed a significant reduction in CD69 expression, while pre-treatment with HOE-140 only decreased CD69 expression in T CD8^+^ cells (Fig. 4 I,L).

**Figure 5:**
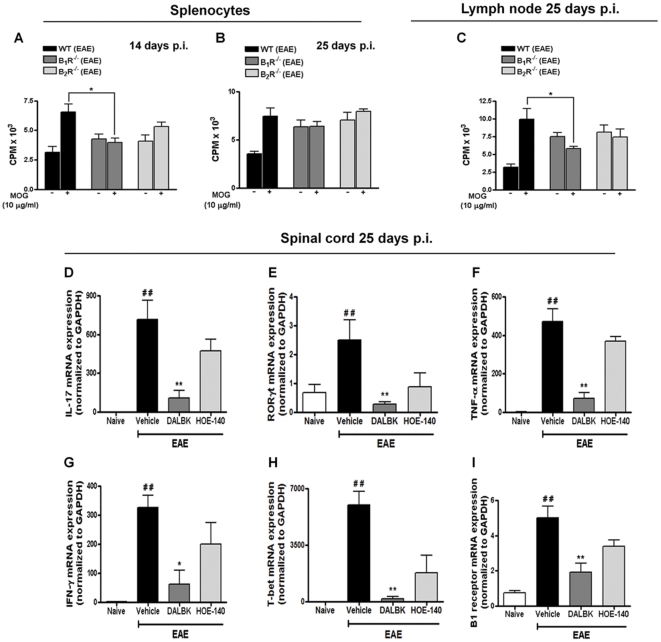
B_1_R inhibition affects the peripheral and central MOG-specific immune responses during EAE pathology. Splenocytes (A, B) and drained lymph node (C) were isolated from immunized mice (EAE group) and B_1_R^−/−^ and B_2_R^−/−^ mice on days 14 and 25 p.i. The cells (1×10^6^/well) were cultured in the presence or absence of MOG_35–55_ (10 µg/ml) for 72 h to assess proliferation by [^3^H] thymidine incorporation. Total RNA was extracted from the lumbar spinal cord of mice in the naive group, the control group (EAE), DALBK (50 nmol/kg) mice, HOE-140 (50 nmol/kg) mice, B_1_R^−/−^ mice and B_2_R^−/−^ mice on day 25 p.i. The mRNA levels of IL-17 (D), ROR-γT (E), TNF-α (F), IFN- γ (G), T-bet (H) and B_1_R (I) were measured. GAPDH mRNA was used to normalize the relative amounts of mRNA. The data are presented as mean ± SEM of six to nine mice/group and are representative of three independent experiments. ^#^P<0.05, ^##^P<0.001 versus the naive group, *P<0.05, **P<0.001 versus the EAE group (one-way ANOVA with the Newmann-Keuls post-hoc test).

A recent study demonstrated that CD4^+^ T cells are activated in the periphery by the MHC class II^+^ APC, which in turn increases the proliferative response and migration to the subarachnoid space, resulting in the formation of large T cell aggregates in the CNS [24]. In order to investigate whether or not B_1_R or B_2_R influences T cell proliferative responses, we evaluated the incorporation of thymidine in lymph node and spleen cells from WT, B_1_R^−/−^ and B_2_R^−/−^ immunized mice. In WT animals with EAE, a significant increase was observed in the proliferative response towards MOG_35–55_ re-stimulation (Fig. 5 A,C), whereas in splenocytes (Fig. 5 A,B) and lymph node cells (Fig. 5C) from B_1_R^−/−^ mice this proliferation was markedly decreased. Taken together, these results suggest that the blockade of B_1_R in the induction phase of EAE modulates the activation and/or differentiation of T_H_1 and T_H_17-MOG reactive cells.

### Inhibition of B_1_R in the induction phase of EAE suppresses T_H_1 and T_H_17 autoimmunity in the CNS

Increasing evidence suggest a role for T_H_17 and T_H_1 in CNS damage, especially the observation of these subpopulations and their signature cytokines in the brains of individuals with MS [25], [26], [27]. The T_H_1 cells are characterized by their expression of IFN-γ and its differentiation is orchestrated by the transcription factor T-bet, whereas T_H_17 mainly produces IL-17 and the transcription factor involved in its differentiation is the retinoid orphan receptor (ROR-γT) [28]. Therefore, we next investigated whether or not the blockade of B_1_R in the induction phase could reduce the expression of IL-17 (Fig. 5D), ROR-γT (Fig. 5E), TNF-α (Fig. 5F), IFN-γ (Fig. 5G) and T-bet (Fig. 5H) in the lumbar spinal cord, 25 days post-immunization. In addition, treatment with DALBK during induction phase of EAE prevented the up-regulation of B_1_R (Fig. 5I).

### Blockade of B_1_R after disease onset reduces clinical symptoms and neuroinflammation induced by the EAE model

**Figure 6:**
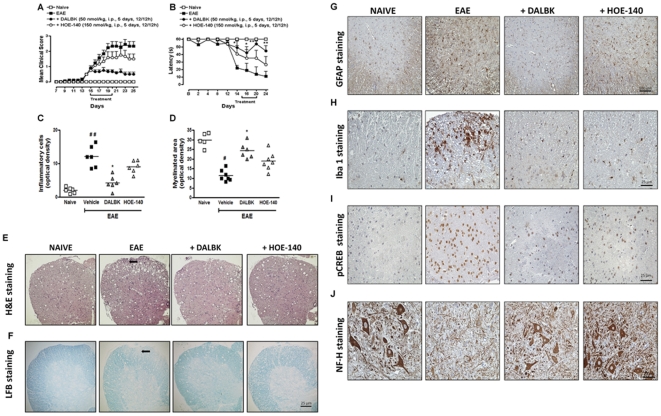
Blockade of kinin B_1_R in the chronic disease phase decreased the clinical symptoms and neuroinflammation in the EAE model. The clinical score (A) and locomotor activity (B) were analyzed 25 days after the animals were immunized with MOG_35–55_ peptide/CFA. The inflammatory infiltrates (H&E staining; C, E), demyelination area (LFB staining; D, F), immunoreactivity of GFAP (G), Iba-1 (H), phospho-CREB (I) and neurofilament-H (J) were analyzed 25 days p.i. in the lumbar spinal cords of mice in the naive group, the control group (EAE) and in mice treated in the chronic phase (days 15–20) with B_1_R (DALBK, 50 nmol/kg, i.p.) or B_2_R (HOE-140, 150 nmol/kg, i.p.) antagonists. Specifically, four alternate 5-µm sections (six to nine animals/group) of the white matter (E–I) and grey matter (J) of the lumbar spinal cord were obtained between L4–L6. Scale bar corresponds to 25 µm and applies throughout. The data are presented as mean ± SEM of six to nine mice/group and are representative of three independent experiments. ^#^P<0.05, ^##^P<0.001 versus the naive group, *P<0.05 and **P<0.001 versus the EAE group (one-way ANOVA with the Newmann-Keuls post-hoc test).

Since MS patients urgently need new and more efficacious therapies to be used after disease outcome, we next investigated whether therapeutic treatment with the B_1_R antagonist DALBK (50 nmol/kg, i.p.) or the B_2_R antagonist HOE-140 (150 nmol/kg, i.p.) might be effective in controlling EAE. The mice were treated for 5 days, twice a day, as soon as clinical signs of the disease appeared (chronic phase: day 15 to day 20 post-immunization). Remarkably, only the therapeutic treatment with DALBK consistently blocked disease progression and motor deficits (Fig. 6 A,B). On day 25, the lumbar spinal cord from animals treated in the chronic phase of EAE with DALBK or HOE-140 showed a significant decrease in levels of cell infiltration (Fig. 6 C,E), demyelination (Fig. 6 D,F), astrocytes (Fig. 6G), microglial activation (Fig. 6H) and CREB phosphorylation (Fig. 6I). In order to further evaluate the loss of spinal cord axons, we assessed the level of neurofilament heavy proteins (NF-H) [29]. In the EAE control group there was a significant loss of axons, while mice treated in the chronic phase of EAE with DALBK (50 nmol/kg, i.p.) or HOE-140 (150 nmol/kg, i.p.) showed a significantly attenuated loss of axons (Fig. 6J). Together, our data suggest that blockade of B_1_R in the CNS showed a dominant role in hampering EAE progression, mainly by affecting the function of astrocytes/microglia, which could lead to neuronal dysfunction.

### The dual role of kinin B_1_R

**Figure 7:**
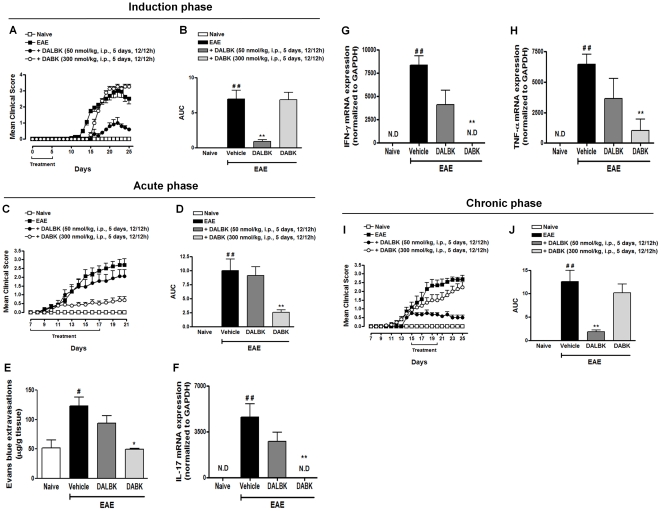
Activation of kinin B_1_R in the acute disease phase improved the pathology of EAE. The clinical score (A, C and I) and area under the curve (AUC) (B, D and J) were analyzed in the induction (day 0–5), acute (day 7–17) and chronic (day 15–20) phases of EAE, respectively. The animals were separated into different groups: naive group, EAE group, mice treated with the B_1_R agonist DABK (300 nmol/kg, i.p.) and mice treated with the B_1_R antagonist DALBK (50 nmol/kg, i.p.). The agonists/antagonists were administered intraperitoneally (i.p.), twice a day (12/12 h), during different time-points of EAE. In the acute phase of EAE, the pharmacological activation of B_1_R (DABK treatment: 300 nmol/kg, i.p.) significantly decreased Evan's blue extravasations in the spinal cord on day 21 compared to the EAE control group (E) and the mRNA levels of IL-17 (F), IFN-γ (G) and TNF-α (H), as measured by RT-PCR. The GAPDH mRNA was used as an endogenous control in the RT-PCR assay. The data are presented as mean ± SEM of six to nine mice/group and are representative of four independent experiments. ^#^P<0.05, ^##^P<0.001 versus the naive group, *P<0.05 and **P<0.001 versus the EAE-treated group (one-way ANOVA with the Newmann-Keuls post-hoc test). N.D. not detected.

So far, our data and other recent reports in the literature have demonstrated a protective role of kinin receptor blockade in EAE progression [13], [14], [15]. However, an unexpected report showed that the activation of B_1_R ameliorates the disease [17]. In order to clarify this discrepancy, we investigated whether or not the B_1_R agonist (DABK) could exert a protective action over EAE throughout the three different stages of the disease (see scheme in Fig. 1A). Treatment with the B_1_R agonist during the induction phase (days 0–5) delayed the onset of clinical signs of EAE by 3 days, as observed previously [17]. However, in our hands the same treatment (DABK, 300 nmol/kg, i.p.) resulted in a severe disease that was similar to the severity of disease observed in the EAE control group (Fig. 7A), and this slight delay did not reach significant difference when calculated based on the area under the curve (Fig. 7B). These results indicate that activation of B_1_R during the EAE induction phase does not improve locomotor activity induced by EAE. This data suggests that in this phase the best therapeutic strategy is the inhibition of B_1_R (Fig. 7 A,B), which corroborates previous data [15].

Corroborating and extending previously published data, the treatment of animals with the selective B_1_R agonist DABK (300 nmol/kg, i.p.) from day 7 to 17 (acute phase) strikingly inhibited EAE progression (Fig. 7 C,D), whereas the B_1_ antagonist DALBK (50 nmol/kg, i.p.) did not alter the course of the disease (Fig. 7 C,D). Herein, in order to elucidate this peculiar data, we considered which process would be more likely to happen in the acute phase of the disease. A pivotal step in triggering CNS inflammation is disruption of the blood-brain barrier (BBB) [30]. In order to investigate whether the activation of B_1_R in the acute phase influenced BBB permeability and neuroinflammation, we used the Evan's blue assay and T_H_1- and T_H_17-cytokine expression in the lumbar spinal cord, respectively. On day 21 after immunization, the EAE group showed a significant increase in Evan's blue extravasations, demonstrating the disruption of BBB permeability (Fig. 7E). Interestingly, the B_1_R agonist DABK (300 nmol/kg, i.p.) markedly reduced Evan's blue extravasations in the spinal cord, whereas the blockade of B_1_R with DALBK induced an increase in the BBB permeability (Fig. 7E). In addition, in the acute phase of EAE, DABK treatment (300 nmol/kg, i.p.) significantly reduced IL-17 (Fig. 7F), IFN-γ (Fig. 7G) and TNF-α (Fig. 7H) mRNA in the spinal cord on day 21. Of note, therapeutic treatment with the B_1_R agonist DABK (300 nmol/kg, i.p.) during the chronic phase of EAE (day 15 to 20) failed to inhibit the clinical score (Fig. 7 I,J) and locomotor deficits (data not shown) induced by EAE. The study of this disease stage has huge importance since therapies applied after disease outcomes are mostly clinically relevant. Our work solved many conflicting data in the literature and indicates that in the chronic phase of EAE the best therapeutic option would be the blockade of B_1_R. Taken together, these results suggest that, in the acute phase of immunization, the kinin B_1_ agonist (DABK, 300 nmol/kg, i.p.) inhibits disruption of the BBB permeability, prevents the entry of both T_H_17 and T_H_1 cells into the CNS and consequently suppresses neuroinflammation. Unlike in the induction and chronic phases, in the acute phase B_1_R activation positively regulates EAE progression, suggesting a dual role of this receptor at different time points of EAE progression.

## Discussion

**Figure 8:**
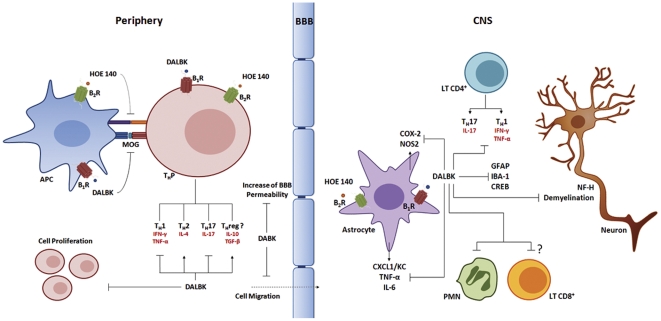
Schematic representation of the mechanism via which kinin regulates the physiopathology of the EAE model. The blockade of kinin B_1_R in either the induction or the chronic phase of EAE suppressed disease progression with the concomitant suppression of T_H_1 and T_H_17-myelin-specific cell development in at least two different stages: (1) during onset of the peripheral immune response, through the modulation of differentiation and/or expansion of auto-aggressive T_H_ cells in the MOG_35–55_-specific immune responses; and (2) during neuroinflammation by affecting the auto-aggressive function of T cells and astrocytes. However, the blockade of B_1_R in the acute phase of EAE only had a slight effect, whereas that of the B_1_ agonist also given at this time markedly reduced disease severity through the inhibition of increased BBB permeability and cell migration, and consequently blocked CNS inflammation. Altogether, we found that kinins, especially B_1_R subtypes, have a dual role during the progression of EAE by distinct mechanisms of action at each stage of disease progression. APC: antigen-presenting cell; DALBK: des-Arg^9^-[Leu^8^]-BK; HOE-140: D-Arg-[Hyp^3^,Thi^5^,D-Tic^7^,Oic^8^]-BK; DABK: des-Arg^9^-BK; MOG: myelin oligodrendrocyte glycoprotein; B_1_R: kinin B_1_ receptor; B_2_R: kinin B_2_ receptor; T_H_P: precursor T cell; BBB: blood-brain barrier; CXCL1/KC: keratinocyte-derived chemokine, TNF-α; tumour necrosis factor-alpha; IFN-γ: interferon-gamma; TGF-β: transforming growth factor beta; PMN: polymorphonuclear leukocytes; GFAP: glial fibrillary acidic protein; Iba1: ionized calcium binding adaptor molecule 1; CREB: cAMP response element-binding; NF-H: neurofilament-H. (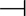), inhibition; 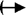), stimulation.

The main results to emerge from the present study are, to the best of our knowledge, the first pieces of evidence to show that the blockade of kinin B_1_R, and, to a lesser extent, the B_2_R subtype, either in the EAE induction or chronic phase, prevented disease progression by modulating the onset of the immune response and by affecting the functioning of astrocytes/microglia cells, respectively. However, we also found that the systemic administration of the B_1_ agonist but not the antagonist, given in the acute phase of the disease, markedly reduced disease severity and inhibited the increase in BBB permeability, blocking neuroinflammation. Our last findings confirm and also extend previous data which indicated that the activation of B_1_R can ameliorate EAE severity by controlling the migration of pro-inflammatory T cells across the BBB into the CNS [17]. Our data provide what we believe is new evidence to demonstrate that, depending on the phase of treatment, either a selective B_1_ agonist or a selective B_1_ antagonist is able to block EAE progression through different mechanisms (see proposed scheme in Fig. 8).

Furthermore, both the genetic and pharmacological data presented in this study showed that blockade of the B_2_R-modulated activation of T CD4^+^ and, particularly, CD8^+^ MOG-reactive cells in the peripheral lymphoid tissue decreased CREB phosphorylation in the spinal cord, decreased neuropathic pain and also attenuated the loss of axons in the CNS. These findings largely extend those of a previous study which indicated that B_2_R^−/−^ mice showed a reduction in the clinical parameters of MOG-induced EAE through the modulation of leukocyte recruitment into the CNS [14]. Nonetheless, unlike with B_1_R, B_2_R inhibition was not able to significantly reduce the size of the demyelination area, nor the central inflammatory cell infiltration, justifying the moderate impact found for all of the parameters of EAE progression studied. These results confirmed the dominant role of B_1_R in the development of EAE [15], [17].

Indeed, it is widely accepted that the activation of both B_1_R and B_2_R positively contribute to pain generation and inflammation [11]. Besides this, many studies imply the involvement of the kallikrein-kinin system in MS and the EAE model [13], [14], [15]. Nonetheless two recent studies using MOG-induced EAE presented contradictory results; i.e., Gobel and collaborators showed that mice with the deletion of B_1_R presented a significantly reduced disease severity compared to the EAE control group, whereas Schulze-Topphoff and colleagues demonstrated a greater clinical disease severity in these animals. The discrepancies between our findings and those reported by Schulze-Topphoff et al. could be explained by certain genetic differences and the method of maintenance of our B_1_R^−/−^ mutant mice, as well as by the gene-environment risk factor. Schulze-Topphoff et al. used B_1_R^−/−^ mice after they were backcrossed to C57BL/6 to produce F_10_ offspring with a SV129 background. Our B_1_R^−/−^ knockout animals were obtained by the same way, although after the F_10_ offspring we maintained the mutant line by crossing them with each other. A necessary caveat with this design is that time, isolation and constant generations among homozygous mutants increase the risk of *de novo* mutations, which could lead to biasing genetic difference between the congenics, the parental recipient strain, and, particularly, the F_10_ offspring breed mutant, which can often characterize an inbred substrain [31], [32], [33]. In addition, some gene-by-environment risk factors may be responsible for the appearance of phenotypic differences between similar strains [34], [35], [36]. Another relevant point is that our animals were immunized twice with MOG on days 0 and 7 in order to increase the incidence of EAE, as previously described (24). However whether the double MOG immunization could have induced some degree of tolerance in our B_1_R^−/−^ mice needs to be further investigated. Here, we hypothesize that this apparent discrepancy could reside in the unusual role played by B_1_R throughout the different phases of EAE progression. We further examined this hypothesis by using a kinin agonist or antagonist at different time points of EAE progression.

Accumulated evidence now suggests that in the induction phase of EAE and MS disease T cells in the periphery become activated by a viral or another infectious antigen [6]. Herein, we show that blockade of kinin B_1_R in the induction phase of EAE consistently inhibited onset of the immune response by modulating the activation of T_H_1 and T_H_17-MOG reactive cells during the presentation of myelin antigens in peripheral lymphoid organs. Consequently, these cells failed to differentiate, proliferate and migrate to the CNS effectively, an effect that abrogated the development of EAE. In fact, our data extends the results of recent reports which indicated that modulation of the microenvironment during antigen presentation and cell activation altered the immune response and then the course of the disease, mainly by diminishing the response of MOG-specific T cells [37], [38], [39]. In agreement with our data, Aliberti et al. showed that kinin can serve as a danger signal that triggers dendritic cell activation, driving T cell polarization, which means that kinins can modulate the immune response at the very beginning of the disease [40].

Another interesting aspect investigated in the present study was the fact that after peripheral activation, CD4^+^ T cells effectively migrated to the CNS during the acute phase of EAE. Here, we found that in the acute phase of EAE, the blockade of B_1_R occurred after the presentation of antigens in peripheral lymphoid organs and, at the same time, treatment of animals with the B_1_ antagonist had no effect. In marked contrast, the activation of B_1_R, assessed via treatment with the B_1_ agonist DABK, inhibited the progression of EAE and neuroinflammation by modulating the permeability of the BBB, a pivotal step that triggers CNS inflammation, an event which is directly related to the development of MS [30], [41]. A recent report showed that in the passive induction of EAE, activation of B_1_R with the agonist R838 (day 0–10) after the adoptive transfer of proteolipid protein (PLP)_139–151_-reactive lymphocytes significantly reduced clinical disease severity when compared with the EAE-control group [17]. Interestingly, it is worth highlighting the fact that the period of treatment of animals in this previous work using the passive induction protocol (day 0 to day 10 after transfer) [17] can be compared with our acute phase of EAE (day 7–17) after the active induction protocol, since the immune response had been already triggered. During the above mentioned period it is possible to evaluate initiation of the myelin-reactive immune attack, as well as T_H_1 and T_H_17 cell migration to the CNS [42]. Based on these results, it is tempting to suggest that activation of B_1_R in the acute phase of EAE seems to affect CNS inflammation and control the migration of pro-inflammatory T cells across the BBB into the CNS, as previously described [17].

Recent data from the literature have shown that bradykinin (BK), a preferential B_2_R agonist, is able to selectively open the BBB, since BK accelerate the release of TNF-α [43], [44]. Of note, in animal models, B_2_R agonist like Cereport® (RMP-7) has been used by the intracarotid route in order to increase the BBB permeability and consequently enhance the delivery of chemotherapeutic agents to the brain tumor area [45], [46], [47]. More recently, a very interesting study conducted by Liu et al. demonstrated that BK increases the BBB permeability by down-regulating the expression levels of tight junction (TJ)-associated proteins, such as zonula occluden-1 (ZO-1), occludin, and claudin-5 and rearranging cytoskeleton protein filamentous actin (F-actin) [48]. However, additional studies are needed to assess the precise mechanisms by which BK modulate the BBB permeability during neuroinflammation conditions, such as MS.

Concerning the chronic phase of EAE, it has been reported that CD4^+^ T cells enter the subarachnoid space by crossing the blood-cerebrospinal fluid (CSF) barrier in either the choroid plexus or in the meningeal venules, and they are re-activated by MHC class II-expressing macrophages/microglia and DCs. Afterwards, macrophage and glial cells secrete soluble mediators that trigger demyelination and attract further inflammatory cells into the CNS [5]. Of note, our data revealed that the blockade of B_1_R in the chronic phase of EAE consistently improved the clinical signs and neuroinflammation induced by EAE. Moreover, *in vitro* pre-treatment with the B_1_R antagonist (DALBK) blocked the pro-inflammatory release/expression induced by IFN-γ in primary astrocytes cultures (data not shown). At this same point in time, due to the peptide nature of these antagonists, it is highly unlikely that, given systemically, they could cross the BBB in opposite directions to block central kinin B_1_R or B_2_R. However, several studies suggested that when under neuroinflammatory conditions, the activation or damage of cellular components of the BBB could facilitate leukocyte infiltration leading to oligodendrocyte death, axonal damage, demyelination and lesion development [30]. Likewise, a recent report showed that B_1_R antagonist (R715) treatment, which started once the animals were already displaying the first clinical symptoms of EAE (chronic phase), markedly inhibited the EAE score [15]. Therefore, we can suggest that after disruption of the BBB in the chronic phase of EAE, the kinin B_1_R antagonists most likely penetrate the CNS and mainly hamper EAE progression by affecting the functioning of astrocytes cells.

Altogether, the present study identified B_1_R as a key mediator in EAE disease and suggested that kinin B_1_R displays a dual role in the progression of EAE via distinct mechanisms at each stage of the disease, mainly through the modulation of T_H_1 and T_H_17-myelin-specific lymphocytes and glial cell-dependent pathways (Fig. 8). Our findings open up important options for the development of clinically relevant therapies for the management of MS, as well as for other immune diseases in which T_H_1 and T_H_17 cells play a key role.

## Materials and Methods

### Experimental animals

The experiments were conducted using female C57BL/6, kinin B_1_R-knockout (B_1_R^−/−^) and kinin B_2_R-knockout (B_2_R^−/−^) mice (6–10 weeks old). The B_1_R gene was cloned from a genomic library of 129/SvJ mice in λFIXII [49]. The targeting vector was generated by flanking the neomycin resistance gene with a 1.0-kb genomic 5′ fragment of the B_1_-coding region and a 7.0-kb 3′ fragment of the B1-coding region. The construct was linearized with NotI and transfected into E14–1 embryonic stem cells by electroporation as previously described [50]. Gancyclovir- and G418-resistant clones were selected and identified by polymerase chain reaction (PCR). Two positive clones were microinjected into C57BL/6 blastocysts, which gave rise to two germ-line chimeras with offspring that were heterozygous for the targeted mutation. The generation of B_2_R mice and gene targeting was performed in the embryonic stem (ES) cell line AB 2.1, derived from 129/SvJ mice [51]. The mouse genomic DNA utilized to construct the targeting vector was obtained from a cosmid clone isolated from a library constructed by Dr John Mudgett (Merck Research Laboratory, Rahway, NJ) from an ES cell line, J1, derived from 129/SvJ mice. The ES cell clones containing the targeted disruption of the B_2_R gene were separated from SNL feeder cells by treating the cell culture with trypsin, allowing the feeder cells to reattach for 30–45 min, and removing the unattached ES cells. Two B_2_R-targeted ES clones, KO-5 and KO-24, were injected into recipient C57BL/6 3-day-old blastocysts; following this, they were re-implanted into day 3 pseudo-pregnant Tac:SW(fBR) mice and allowed to develop to term. The B_1_^−/−^ and B_2_^−/−^ mice used in the experiments originated from ten generations of backcrossing of mice with an initially mixed genetic background (129/SvJ and C57BL/6) with C57BL/6 mice (Taconic, Germantown, NY). Following the F_10_ offspring the line is 99% genetically identical to the recipient strain (C57BL/6) and is considered congenic with it; we maintained this linage by crossing them with each other. The C57BL/6 animals were used as controls. The mice were kept in groups of six to nine animals per cage, maintained under controlled temperature (22±1°C) and humidity (60–80%) conditions with a 12 h light/dark cycle (lights on at 7:00 a.m.) and were given free access to food and water. All procedures used in the present study followed the Guide for the Care and Use of Laboratory Animals (NIH publication No. 85-23) and were approved by the Animal Ethics Committee of the Universidade Federal de Santa Catarina (CEUA-UFSC, protocol number 23080038266/2008-43).

### Drug treatment protocol

The following series of experiments were designed to evaluate how kinin receptors regulate some of the biological processes that occur in the EAE model. (1) In order to determine the effect of the kinin receptor on the origin of the autoimmune response induced by EAE (Induction phase), different groups of animals were treated with the selective B_1_R and B_2_R antagonists and/or agonists twice a day, for 5 days (starting on day 0 until day 5 post-immunization). (2) In order to assess the ability of kinin B_1_R to modulate the development of neuroinflammation induced by EAE (Acute phase), the animals were treated for 10 days (starting on day 7 until day 17 post-immunization). (3) In order to evaluate the effect of B_1_R and B_2_R after the clinical signs of EAE had already been observed (Chronic phase), the animals were treated for 5 days as soon as the first clinical signals appeared (from day 15 to 20 post-immunization). The B_1_R antagonist Des-Arg^9^-[Leu^8^]-BK (DALBK, 50 nmol/kg), the B_1_R agonist Des-Arg^9^-BK (DABK, 300 nmol/kg) or the B_2_R antagonist HOE-140 (HOE, 150 nmol/kg), or their vehicles, were administered intraperitoneally (i.p.) The choice of the dose for each drug was based on pilot experiments (data not shown) or on data previously described in the literature [8], [52].

### EAE induction

The experimental autoimmune encephalomyelitis (EAE) was induced by subcutaneous (s.c.) immunization into the flanks with 200 µl of an emulsion containing 200 µg of MOG_35–55_ peptide and 500 µg of *Mycobacterium tuberculosis* extract H37Ra (Difco Laboratories, Detroit, MI, USA) in incomplete Freund's adjuvant oil (Sigma Chemical Co., St. Louis, MO, USA). This procedure was repeated after 7 days in order to increase the incidence of EAE, as previously described [42]. In addition, the animals received 300 ηg of pertussis toxin (Sigma Chemical Co., St. Louis, MO, USA) i.p. on day 0 and on day 2 post-immunization. Non-immunized (naive) and EAE-group animals were used as the control groups. The animals were monitored daily and neurological impairment was quantified using a clinical scale after day 7 post-immunization [42]. Mice were weighed and observed daily for clinical signs of EAE for up to 25 days post-immunization. Clinical signs of EAE were assessed according to the following scores: 0, no signs of disease; 1 loss of tone in the tail; 2 hindlimb paresis; 3 hindlimb paralysis; 4 tetraplegia; 5 moribund and/or death.

### Behavioural experiments

#### Rotarod test

As a test of locomotor activity and coordination, the mice were placed on a rotarod apparatus at a fixed rotational speed of 4 rpm. The maximum time for each trial was set at 60 s. Rotarod training was performed prior to disease induction and consisted of three consecutive trials in which the animals became familiar with the task. After disease induction, the mice were tested every two days, until day 25 post-immunization.

### Measurement of cytokine production in lymph node (LN) and spleen cells

Inguinal LN cells and splenocytes from the mice were prepared on day 25 after immunization. Briefly, lymph nodes and the spleen were individually macerated in RPMI 1640 medium supplemented with 10% foetal bovine serum, HEPES (20 nM), 2-mercapto ethanol, penicillin (100 U/ml) and streptomycin (100 µg/ml) and the cell suspension was filtered through a 70 µm filter. The resulting suspension was centrifuged at 1500 *g* for 7 min at 4°C. For the spleen tissue, after the initial centrifugation the supernatant was discarded and the cell pellet resuspended in ammonium chloride potassium carbonate buffer (ACK lysis buffer) using 1 ml per donor mouse to lyse red blood cells and incubated on ice for 5 min. After incubation, 9 ml of the medium was added to stop the cell lysis. The cell debris was allowed to settle on the bottom of the tube for 2 min before being transferred to a new 15 ml conical tube and centrifuged for 5 min at 500 *g* and 4°C. Finally, the supernatant was discarded and the cells were resuspended in 2 ml of the medium. Lymph node cells and splenocytes (1×10^6^/well) were cultured in the presence of MOG_35–55_ (10 µg/ml) or in the medium alone. The LN cells and splenocytes were incubated for 48 h at 37°C in a humidified 5% CO_2_ atmosphere. The culture supernatants were collected and stored at −70°C until further analysis. The levels of tumour necrosis factor-α (TNF-α), interferon-γ (IFN-γ), interleukin-17 (IL-17) and interleukin-4 (IL-4) were measured using ELISA kits, according to the manufacturer's recommendations. After collection of the culture supernatants, the cells were used in a flow cytometry assay.

### Proliferation assays

Lymph node and spleen cells (1×10^6^/well) prepared from immunized mice (EAE control group) and kinin B_1_R and B_2_R-knockout mice were cultured in 96-well flat-bottomed microculture plates in the presence of MOG_35–55_ (10 µg/ml) or in medium alone. After 60 h, 0.5 µCi per well [^3^H] of thymidine was added to each well and then incubated for 12 h. The cells were then harvested onto glass fibre filters, and radioactivity was measured in a Liquid Scintillation/Beta Counter LS5000TD (Beckman Coulter, Inc., Brea, CA, USA).

### Flow cytometric analysis of lymphocytes

After incubation with MOG_35–55_, the LN cells were washed with RPMI 1640 medium supplemented with 10% foetal bovine serum and further incubated with the following antibodies for 20 min at 4°C: anti-CD4-PerCP (clone RM4-5, Caltag Laboratories, Burlingame, CA, USA), anti-CD8a-APC (clone 5H10, Caltag Laboratories, Burlingame, CA, USA) and anti-CD69-PE (clone H1.2F3, BD Pharmingen™, San Jose, CA, USA). The data were collected using FACSCanto II (BD Biosciences) and analysed using FlowJo (version 7.5) software.

### Histological analysis

Twenty-five days after the induction of EAE, the mice were deeply anesthetized with 7% chloral hydrate (8 ml/kg; i.p.) and intracardially perfused with 4% of the paraformaldehyde fixative solution in 0.1 M phosphate buffer (pH 7.4). The spinal cords were removed and post-fixed for 24 h in the same solution, and then embedded in paraffin after dehydration and diaphanization. The histological analysis of inflammation and demyelination was performed using haematoxylin-eosin (H&E) and luxol fast blue (LFB), respectively. The settings used for image acquisition were identical for both control and experimental tissues. Four ocular fields per section (six to nine mice per group) were captured and a threshold optical density that best discriminated the nuclear staining of inflammatory cells (haematoxylin-eosin) or myelin (luxol fast blue) was obtained using the NIH ImageJ 1.36b imaging software (NIH, Bethesda, MD, USA) and applied to all experimental groups The total pixel intensity was determined, and the data are expressed as optical density (O.D.).

### Immunohistochemistry assay

Immunohistochemical analysis was performed on paraffin-embedded lumbar spinal cord tissue sections (5 µm) using monoclonal mouse anti-CD3^+^ T cells (1∶100), monoclonal mouse anti-GFAP (1∶300), polyclonal goat anti-Iba1 (1∶200), monoclonal mouse anti-neurofilament H (1∶200) and polyclonal rabbit anti-phospho-CREB (1∶300), according to the method previously described [53]. After quenching of endogenous peroxidase with 1.5% hydrogen peroxide in methanol (v/v) for 20 min, high-temperature antigen retrieval was performed by immersing the slides in a water bath at 95 to 98°C in 10 mmol/l trisodium citrate buffer, pH 6.0, for 45 min. The slides were then processed using the Vectastain Elite ABC reagent (Vector Laboratories, Burlingame, CA), according to the manufacturer's instructions. Following this the immune complexes were visualized with 0.05% 3,3′-diaminobenzidine tetrahydrochloride (DAB: Dako Cytomation, Glostrup, Denmark) plus 0.03% H_2_O_2_ in PBS (for exactly 1 min). The reaction was stopped by thorough washing in water and counterstained with Harris's haematoxylin. Besides staining untreated animals as negative controls, sections were also incubated without the primary antibody (data not shown), and these controls resulted in little or no staining. To eliminate temporal variations, control and experimental tissues were placed on the same slide and processed under the same conditions. Images were acquired using a Sight DS-5M-L1 digital camera connected to an Eclipse 50i light microscope (both from Nikon, Melville, NY, USA). Specifically, four alternate 5-µm sections of the lumbar spinal cord with an individual distance of 150 µm were obtained between L4 and L6. Images of stained white matter of the spinal cord were acquired using a Sight DS-5M-L1 digital camera (Nikon, Melville, NY, USA) connected to an Eclipse 50i light microscope (Nikon). For the NF-H analysis, images of the grey matter of the lumbar spinal cord (dorsal and ventral horns) were obtained. The optical density threshold that best discriminated staining from the background was selected using the “Threshold Color” plug of NIH ImageJ 1.36b imaging software (NIH, Bethesda, MD, USA) and applied to all experimental groups. For phospho-CREB, GFAP, Iba-1 and NF-H analyses, the total pixel intensity was determined and the data were expressed as optical density (O.D.). In order to analyse the number of T lymphocyte cells, CD3-positive cells were visually inspected by counting the number of labelled cells in the white matter of the lumbar spinal cord section, using a counting grid at a ×400 magnification.

### Real-time quantitative PCR

Lumbar spinal cord (six to nine animals/group) tissues were removed 25 days post-immunization and the total RNA was extracted using the Trizol protocol. The reverse transcription assay was carried out as described in the M-MLV Reverse Transcriptase (Invitrogen, Carlsbad, CA, USA), according to the manufacturer's instructions. Real-time quantitative PCR analysis of mRNA was performed in StepOnePlus™ using the TaqMan® Universal PCR Master Mix Kit (Applied Biosystems, Foster City, CA, USA) for quantification of the amplicons, and 100 ng of cDNA were used in each reaction. The cDNA was amplified in triplicate using specific TaqMan Gene Expression target genes, the 3′ quencher MGB and FAM-labelled probes for TNF-α (Mm00443258_m1), IFN-γ (Mm99999071_m1), interleukin-17 (IL-17) (Mm00439618_m1), kinin B_1_R (Mm00432059_s1), ROR-γT (Mm00441144_g1), T-bet (Mm00450960_m1) and GAPDH (NM_008084.2), which were obtained from Applied Biosystems (Foster City, CA, USA). The thermocycler parameters were as follows: 50°C for 2 min, 95°C for 10 min, 50 cycles of 95°C for 15 s, and 60°C for 1 min. Samples without cDNA were analysed as “no template” controls. The mRNA levels were quantified using the comparative threshold cycle (Ct) method [54], where the mean Ct values from duplicate measurements were used to calculate the expression of the target gene, with normalization to the housekeeping gene GAPDH in the samples.

### Measurement of BBB permeability

The BBB permeability was assessed by measuring the extravasations of Evan's blue (EB) dye, as previously described [55]. Briefly, on day 21, the mice were i.v. injected with 0.1 ml 2% EB, which was allowed to circulate for 60 min. Following this period of circulation, the animals were transcardially perfused with 0.9% phosphate buffered saline (PBS) to remove the intravascular EB dye. The lumbar spinal cord (L4–L6) was further dissected and weighed. Tissue was incubated in 600 µl formamide at 60°C for 24 h. At the end of incubation, the tissues were removed and the formamide solution was centrifuged at 20,000 *g* for 20 min. The supernatant solution was collected and the optical density was measured at 620 nm to determine the relative amount of EB dye in each sample.

### Materials

Pertussis toxin, PBS, H&E, H_2_O_2_, Incomplete Freund's adjuvant oil, kinin B_1_R antagonist des-Arg^9^-[Leu^8^]-BK, kinin B_1_R agonist des-Arg^9^-BK, HEPES, penicillin, streptomycin, Na-EDTA, trypsin, deoxyribonuclease I (DNase) and bovine serum albumin (BSA) were purchased from Sigma Chemical Co. (St. Louis, MO, USA). The kinin B_2_R antagonist HOE-140 was donated by Aventis (Frankfurt Main, Germany). Formaldehyde, formamide, NH_4_Cl and KHCO_3_ were obtained from Merck (Frankfurt, Darmstadt, Germany). The MOG_35–55_ peptide (MEVGWYRSPFSRVVHLYRNGK) was obtained from EZBiolab, Carmel, IN 46032, USA; and *M. tuberculosis* extract H37Ra from Difco Laboratories, Detroit, MI, USA. The RPMI 1640 and foetal bovine serum were purchased from GIBCO (Carlsbad, CA, USA). [^3^H] Thymidine (0.5 mCi/ml) was provided by Amersham Biosciences (Piscataway, NJ, USA). The anti-mouse-TNF-α, IFN-γ, IL-17 and IL-4 DuoSet kits were obtained from R&D Systems. The monoclonal mouse anti-CD3^+^ T cells were purchased from Santa Cruz Biotechnology (Santa Cruz, CA, USA) and the polyclonal goat anti-ionized calcium binding adaptor molecule 1 (Iba-1) was purchased from Abcam (Cambridge, MA, USA). The monoclonal mouse anti-astrocytes marker glial fibrillary acidic protein (GFAP), monoclonal mouse anti-neurofilament H (NF-H) and polyclonal rabbit anti-nuclear phospho-cyclic AMP response element binding protein (CREB) were purchased from Cell Signaling Technology (Danvers, MA, USA). Secondary antibody Envision Plus, streptavidin-HRP reagent and 3,3-diaminobenzidine chromogen were purchased from Dako Cytomation (Carpinteria, CA, USA). The primers and probes for mouse TNF-α, IFN-γ, IL-17, kinin B_1_R, ROR-γT, T-bet and GAPDH were purchased from Applied Biosystems (Warrington, UK). The other reagents used were of analytical grade and obtained from different commercial sources.

### Statistical analysis

All data are presented as mean ± SEM of six to nine mice/group and are representative of three/four independent experiments. A statistical comparison of the data was performed by two-way ANOVA followed by Bonferroni's post-hoc test or one-way ANOVA followed by Bonferroni's or Newman-Keuls's test, depending on the experimental protocol; *p*-values less than 0.05 (*p*<0.05 or less) were considered significant. The statistical analyses were performed using GraphPad Prism 4 software (GraphPad Software Inc., San Diego, CA, USA).
